# Genetic Background Analysis of Protein C Deficiency Demonstrates a Recurrent Mutation Associated with Venous Thrombosis in Chinese Population

**DOI:** 10.1371/journal.pone.0035773

**Published:** 2012-04-24

**Authors:** Liang Tang, Tao Guo, Rui Yang, Heng Mei, Huafang Wang, Xuan Lu, Jianming Yu, Qingyun Wang, Yu Hu

**Affiliations:** 1 Institute of Hematology, Union Hospital, Tongji Medical College, Huazhong University of Science and Technology, Wuhan, Hubei, People's Republic of China; 2 Hubei Clinical and Research Center of Thrombosis and Hemostasis, Wuhan, Hubei, People's Republic of China; 3 Targeted Biotherapy Key Laboratory of Ministry of Education, Wuhan, Hubei, People's Republic of China; Leiden University Medical Center, The Netherlands

## Abstract

**Background:**

Protein C (PC) is one of the most important physiological inhibitors of coagulation proteases. Hereditary PC deficiency causes a predisposition to venous thrombosis (VT). The genetic characteristics of PC deficiency in the Chinese population remain unknown.

**Methods:**

Thirty-four unrelated probands diagnosed with hereditary PC deficiency were investigated. PC activity and antigen levels were measured. Mutation analysis was performed by sequencing the *PROC* gene. *In silico* analyses, including PolyPhen-2, SIFT, multiple sequence alignment, splicing prediction, and protein molecular modeling were performed to predict the consequences of each variant identified. One recurrent mutation and its relative risk for thrombosis in relatives were analyzed in 11 families. The recurrent mutation was subsequently detected in a case (VT patients)-control study, and the adjusted odds ratio (OR) for VT risk was calculated by logistic regression analysis.

**Results:**

A total of 18 different mutations, including 12 novel variants, were identified. One common mutation, *PROC* c.565C>T (rs146922325:C>T), was found in 17 of the 34 probands. The family study showed that first-degree relatives bearing this variant had an 8.8-fold (95%CI = 1.1–71.6) increased risk of venous thrombosis. The case-control (1003 vs. 1031) study identified this mutation in 5.88% patients and in 0.87% controls, respectively. The mutant allele conferred a high predisposition to venous thrombosis (adjusted OR = 7.34, 95%CI = 3.61–14.94). The plasma PC activity and antigen levels in heterozygotes were 51.73±6.92 U/dl and 75.17±4.84 U/dl, respectively.

**Conclusions:**

This is the first study on the genetic background of PC deficiency in the Chinese population. The *PROC* c.565C>T mutation is the most frequent cause of PC deficiency as well as a prevalent risk factor for VT in Chinese individuals. The inclusion of this variant in routine thrombophilic detection may improve the diagnosis and prevention of venous thrombosis.

## Introduction

Protein C, the key component of the PC anticoagulant system, is an important vitamin K-dependent protein that regulates the physiological coagulation cascade by inactivating factors Va and VIIIa upon activation by thrombin [1]–[3]. The mature PC molecule is a single- chain, 62-kDa glycoprotein that is synthesized by hepatocytes as a 461-amino acid precursor from which a 42-amino acid signal peptide is cleaved. Protein C is composed of a γ-carboxy-glutamic acid residue (Gla) domain, two epidermal growth factor (EGF)-like domains, a short activation peptide, and a serine protease domain [4], [5]. Thrombin cleaves PC at Arg169, removes the activation peptide and generates activated protein C (APC) [6]. In addition to its anticoagulant properties, APC has anti-inflammatory and cytoprotective functions, which are exerted when APC activating protease activated receptor-1 (PAR-1) [7], [8]. The human protein C gene (*PROC*) is located on chromosome 2q13-q14 and comprises nine exons spanning 11 kb [9], [10].

The most common genetic risk factors for venous thrombosis in Whites, factor V Leiden (FV R506Q) and prothrombin G20210A polymorphism, are rare in Eastern populations [11]–[13]. Therefore, the three main physiological anticoagulant (antithrombin, protein C, and protein S) deficiencies are important risk factors in Asians, and their diagnosis is of great clinical interest [14], [15]. Hereditary protein C deficiency (OMIM#176860) is usually inherited as an autosomal dominant trait and is associated with an increased risk of venous thrombosis and hereditary thrombophilia. Heterozygous individuals have an approximately 7-fold increased risk of venous thrombosis compared with normal individuals [16]. The homozygous (or compound heterozygous) state of protein C deficiency is much more rare [17]. The overlap in the plasma protein C levels between healthy individuals and PC heterozygotes makes the discrimination between these two groups solely based on a single plasma measurement challenging [18]. Genetic determination is likely to be more powerful.

To date, a number of mutations in the *PROC* gene associated with PC deficiency have been identified, but only a few of these variants were observed in the Chinese population, and the genetic characteristics of PC deficiency in Chinese patients have not been studied. Here, we report the results of the genetic investigation of protein C deficiency in 34 Chinese families and present a prevalent causative variant for PC deficiency and venous thrombosis.

## Methods

### Ethics Statement

This study was approved by the ethics committee of Union Hospital, Huazhong University of Science and Technology and complied with the principles expressed in the Declaration of Helsinki. Written informed consent was obtained from all participants.

### Protein C-deficient patients and sample collection

A total of 34 unrelated probands diagnosed with hereditary protein C deficiency and registered at the Hubei Clinical and Research Center of Thrombosis and Hemostasis from 2008 to 2010 were investigated. The diagnosis was based on repeated low plasma protein C activity measurements and a personal or family history of symptomatic thromboembolic diseases.

Blood samples were collected from all subjects during a non-acute phase of VT (at least one month after a VT incident), and the patients had not received anticoagulant therapy for at least two weeks before the collection. The blood sample was collected by venipuncture into a vacutainer tube containing 1/10 volume of 0.105 mol/L trisodium citrate and was immediately centrifuged at 2000× *g* for 15 min. The platelet-poor plasma was stored at −80°C until assayed. High molecular weight genomic DNA was isolated from white blood cells and standardized to 50 ng/µl.

### Coagulation assays

Protein C, protein S, and antithrombin activities were assayed on a Sysmex CA 7000 Analyzer (Sysmex, Japan) using commercial reagents obtained from Dade Behring-Siemens Healthcare Diagnostics, Germany. Protein C and antithrombin activities were measured using a chromogenic substrate method. The APC-cofactor activity of protein S was evaluated using a clotting method. Protein C antigen was further tested by an enzyme-linked immunosorbent assay using ZYMUTEST Protein C (Hyphen BioMed, France). The normal ranges of these tests in our lab were established in 78 healthy subjects.

### The *PROC* gene analysis

The *PROC* gene was analyzed in each proband by PCR and resequencing. All nine exons, including at least 100 bp flanking intron regions, and the 5′- and 3′-untranslated regions of the human protein C gene were amplified. Detailed amplification conditions and the sequences of the oligonucleotide primers used are available upon request. The amplified fragments were sequenced on an ABI PRISM 3730XL automated sequencer (Applied Biosystems). Identified variants were confirmed on a second PCR product, sequencing on both strands. The variants were designated according to current nomenclature and the recommendations of the Human Genome Variation Society (HGVS, http://www.hgvs.org/mutnomen/) and were checked using the Mutalyzer program [19]–[21]. All new data have been deposited in GenBank under accession numbers NM_000312.3 and NP_000303.1. Each novel variant was then detected in 50 normal individuals (100 alleles) using direct sequencing.

### *In silico* analysis of novel amino acid changes

The possible impact of novel coding sequence changes (amino acid substitutions) on the structure and function of PC was assessed using two bioinformatics tools, Sorting Intolerant From Tolerant (SIFT, http://sift.jcvi.org) and Polymorphism Phenotyping-2 (PolyPhen-2, http://genetics.bwh.harvard.edu/pph2/) [22], [23]. The UniProtKB Protein ID “P04070” and Protein Ensembl ENSP ID “ENSP00000234071” were used, respectively.

The conservation of the affected amino acids were further checked by multiple sequence alignment (HomoloGene, http://www.ncbi.nlm.nih.gov/sites/entrez) with sequences from *Pantroglodytes*, *Canis lupus familiaris*, *Bos taurus*, *Mus musculus*, *Rattus norvegicus*, and *Gallus gallus*.

### Splicing efficiency prediction

The effect of exon-intron boundary variants on the efficiency of splice processing was predicted by two web server programs: SplicePort (http://spliceport.cs.umd.edu/) and Alternative Splice Site Predictor (ASSP, http://www.es.embnet.org/~mwang/assp.html) [24], [25].

### Protein molecular modeling

The molecular structures of the wild-type and mutant proteins were modeled using Swiss model workspace (http://swissmodel.expasy.org/) [26] and noc-3.01 software based on the tridimensional crystal structure of PC (PDB ID: 3F6U) [27].

### Recurrent mutation detection in venous thrombosis patients and controls

The *PROC* c.565C>T mutation, which was common among the probands, was subsequently detected in an independent case-control study to evaluate the OR of this variant for venous thrombosis in the Chinese population.

This secondary study included consecutive unrelated patients (n = 1003) who were diagnosed with venous thrombosis at the Hubei Clinical and Research Center of Thrombosis and Hemostasis from 2008 to 2011. The thrombosis incidents were validated based on clinical manifestations, D-dimer levels, Doppler ultrasound (for deep vein thrombosis), ventilation perfusion lung scan, and/or computed tomography angiography (for pulmonary embolism). Age- and sex-matched controls (n = 1031) without an individual or family history of VT were enrolled from a community screening program in the same regions during the same time window. Demographic data and acquired thrombotic risk factors were recorded. Blood samples were collected from all participants.

All of the participants included in the case-control study were central Chinese. To test whether this PC variant was also common in populations from other regions of China, we recruited a cohort of 492 healthy individuals (43.9% female) who were evaluated for routine physical check-up in Shanghai (Dongfang Hospital, eastern China) in February 2012. The healthy Shanghai participants ranged in age from 38 to 66 years and had no family history of thrombosis. Blood samples were collected, and genomic DNA was extracted and prepared for genotyping.

The c.565C>T mutation was genotyped using the PCR-RFLP method. The mutagenic primers 5′-TCCTTGAACCCTGCACTGTGGCAA-3′ and 5′-TTTCAGGTGACT*A*CGC- TTCTTCTCCAT*G*C-3′ were employed. The underlined and italicized base G in the lower primer was used to introduce a Hin6 I restriction site into the normal allele. In contrast, the underlined and italicized base A was used to eliminate an adjacent restriction site. Therefore, only the PCR product of the common allele has a Hin6 I recognition site. Altogether, 10 µl of PCR product was digested with 5 U of Hin6 I (Fermentas, Canada) for 3 hours at 37°C and then subjected to 2% agarose gel electrophoresis. To verify the genotyping results determined by PCR/RFLP assay, up to 192 DNA samples were randomly selected and subjected to sequencing.

### Family analysis of the recurrent mutation

To avoid bias, probands were excluded, and analyses were performed on first-degree relatives only. Included families were composed of at least two family members (one c.565C>T carrier and one non-carrier). Six families were excluded because they did not meet the criteria. In total, 49 family members from 11 families were enrolled to estimate the thrombotic risk associated with the recurrent mutation. The annual incidence of thrombosis was calculated by dividing the number of events in each group (carriers and non-carriers) by the total number of patient-years of follow-up. For each individual, years of follow-up were defined as the time from the date of birth to either the date of the first episode of venous thrombosis, if any, or December 2011. The relative risks were calculated using only the first thrombotic event during follow-up.

### Statistical analysis

For continuous variables, differences between groups were analyzed by a Student's *t*-test or Mann-Whitney U-test, depending on the normality of the data. A chi-squared test was used for categorical variables. Deviations from Hardy-Weinberg expectations were assessed using both Fisher's Exact test and chi-squared test. Multivariate logistic regression analysis was used to calculate the OR of the c.565C>T mutation for venous thrombosis adjusted for selected confounders (age, gender, smoking status, alcohol abuse, malignant tumor, type 2 diabetes, sedentariness/immobilization, and pregnancy/puerperium). A Cox regression analysis was performed to estimate the relative risk (hazard ratio) for thrombosis in first-degree relatives with the c.565C>T mutation, adjusted for age and gender. Statistical power was estimated using the Power and Sample (PS) size calculation program [28]. A two-tailed *P*<0.05 was considered statistically significant. Analyses were performed using SPSS version 12.0 software (SPSS Inc., Chicago, IL).

## Results

### Mutation profiles

Eighteen different genetic variants (12 novel) were identified in 32 of the 34 probands (94.1%). No candidate mutations were found in the other two subjects, most likely due to gross insertion/deletion of the *PROC* gene. The mutation profiles consisted of 14 missense mutations, two splice site mutations, one small deletion, one small duplication, and one 3′-untranslated region variant. Each novel variant identified in a proband was shown to cosegregate with a low plasma PC level in the family. All of the novel variants were absent in all 50 healthy individuals, suggesting that they are not common polymorphisms. The mutation profiles are summarized in Table 1.

**Table 1:** Mutation profiles, laboratory, and clinical data of for patients with PC deficiency.

Patient No.	Age	Clinical data	PC∶A (U/dl)	PC∶Ag (U/dl)	NT exchange	AA substitution	Ref.
PC1	15	DVT	6.3	12.6	c.889G>C c.1258G>T	p.Asp297His p.Val420Leu	novel novel
PC3	64	DVT	40.4	48.3	c.*73C>T	-	novel
PC4	33	DVT	52.9	58.8	c.524G>A	p.Cys175Tyr	novel
PC6	32	DVT/MVT	36.4	49.2	c.716dupG	p.Ala240GlyfsX17	novel
PC7	55	DVT	52.1	31.5	c.632G>A	p.Arg211Gln	[32]
PC11	24	DVT/PE	9.9	9.2	c.349_352del c.541T>G	p.Phe118AlafsX16 p.Phe181Val	Novel [33]
PC12	45	DVT	61.7	53.4	c.400+5G>A	-	[29]
PC15	65	DVT	49.3	61.7	not found	-	-
PC16	46	DVT	44.1	53.3	c.316T>G	p.Cys106Gly	novel
PC17	46	DVT/PE	57.7	65.2	c.889G>C c.891C>T	p.Asp297His	novel novel
PC19	44	DVT	30.4	76.7	c.935C>T	p.Ser312Leu	[34]
PC20	37	DVT	40.9	38.3	c.658C>T	p.Arg220Trp	[29]
PC22	49	DVT	48.5	53.3	not found	-	-
PC25	42	DVT/AIS	39.4	26.7	c.541T>G c.980A>T	p.Phe181Val p.Glu327Val	[33] novel
PC27	46	DVT	48.7	42.4	c.208A>G	p.Lys70Glu	novel
PC28	54	DVT	40.0	53.0	c.237+5G>A	-	novel
PC29	48	DVT	60.0	88.1	c.669C>A	p.Ser223Arg	novel
PC31	64	DVT	44.2	42.4	c.541T>G	p.Phe181Val	[31]
others	38–67	DVT	40.4–62.3	68.7–83.0	c.565C>T	p.Arg189Trp	[34]

Mutations were designated according to the HGVS nomenclature for variants, comparing with the NCBI Reference Sequences NM_000312.3 and NP_000303.1. Patients with two nucleotide substitutions indicated compound heterozygotes. NT = nucleotide. AA = amino acid. Ref. = References. DVT = deep vein thrombosis. MVT = mesenteric vein thrombosis. PE = pulmonary embolism. AIS = acute ischemic stroke.

### Missense mutations

**Figure 1:**
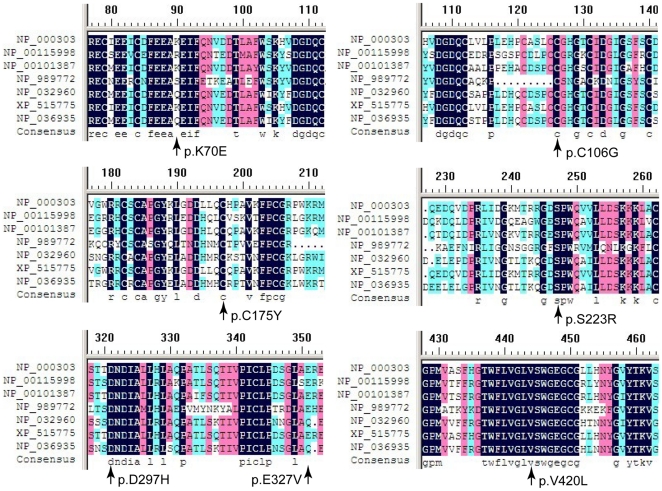
Multiple sequence alignment highlighting the affected amino acids among selected species. The conservation degree of the affected amino acids was evaluated in sequences from *Homo sapiens* (NP_000303.1), *Bos taurus* (NP_001159984.1), *Canis lupus familiaris* (NP_001013871.1), *Gallus gallus* (NP_989772.1), *Mus musculus* (NP_032960.3), *Pan troglodytes* (XP_515775.3), and *Rattus norvegicus* (NP_036935.1).

**Figure 2:**
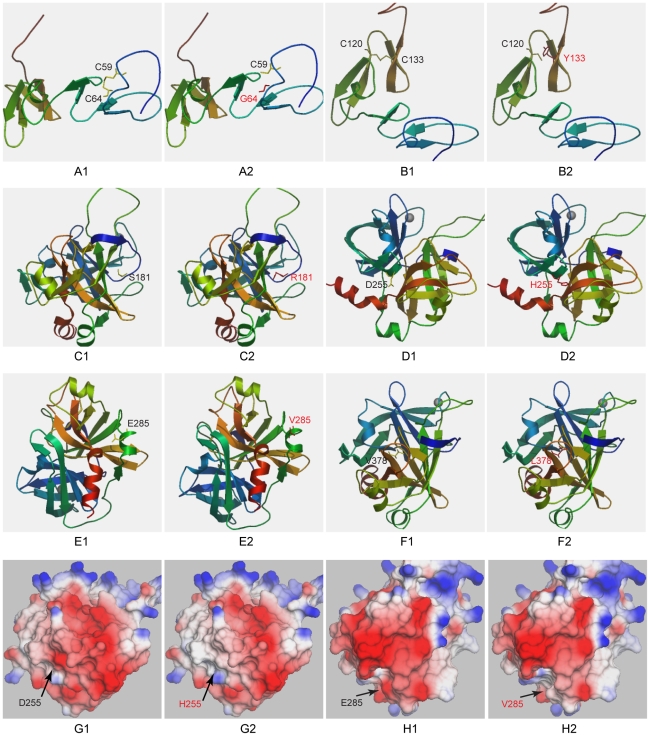
Structure models of the wild-type and mutant PC proteins. The amino acid number was designated according to the previous nomenclature described in the Human Gene Mutation Database. That is, the first 42 amino acids (signal peptide) is subtracted. The wild-type amino acids are shown in yellow and the mutants are shown in red. The D255H and the E285V mutations are also displayed in a solid surface model (G, H) in which the electrostatic potential is clearly indicated.

A total of 14 missense mutations resulted in amino acid substitutions. Eight are reported for the first time here. A multiple sequence alignment among selected species showed that seven of the newly identified mutations led to substitutions of highly conserved amino acids (Figure 1). Although Lys70 (basic amino acid) is not conserved, Glu (acidic amino acid) is never present at this site in the evaluated species. Novel missense mutations of p.Cys175Tyr, p.Asp297His, and p.Val420Leu were predicted to be deleterious by both the SIFT and PolyPhen-2 algorithms. The other four mutations (p.Lys70Glu, p.Cys106Gly, p.Ser223Arg, and p.Glu327Val) were predicted to be damaging by one algorithm. The location of each affected amino acid and the correlative structure of mutant PC are depicted in Figure 2.

### Null mutations

Five of the detected variants were suspected to result either in the complete absence of the gene product or in the expression of a non-functional protein; these mutations were defined as null mutations, and four of them are novel. The frameshift mutations c.719dupG and c.349_352del might produce a premature termination codon and lead to the production of a truncated protein. The c.237+5G>A mutation occurs in the donor splice site of intron C. This substitution changed the splice site scores to 0.000 from original scores of 7.137 and 0.180 assigned by the splicing efficiency prediction programs ASSP and SplicePort, respectively. The closest candidate splice site was 46 bp downstream from the normal splice site. No matter whether the cryptic splice site would be used, this should result in an abnormal translation product. The c.*73C>T variant, 73 bp downstream from the stop codon, completely cosegregated in the PC3 family and was absent in 50 control subjects. This mutation might affect the stability of the PC mRNA.

### Recurrent mutation detection in patients and controls

**Figure 3:**
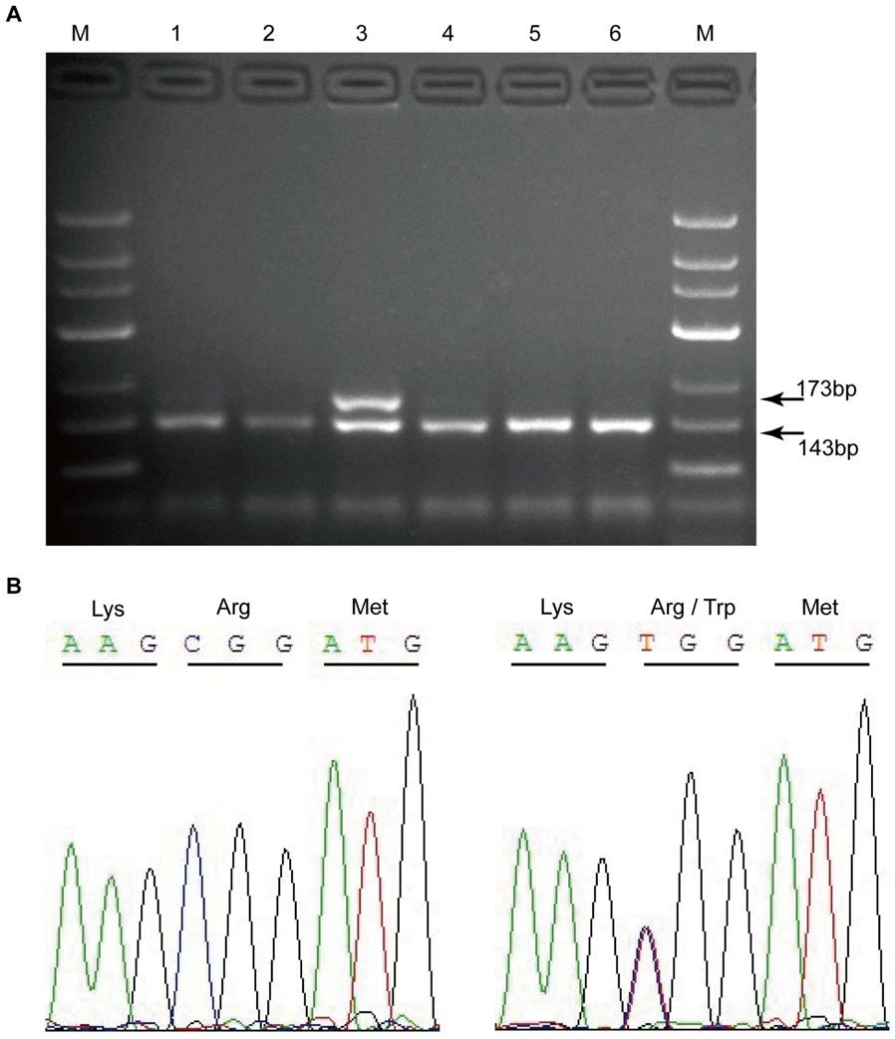
*PROC* c.565C>T variant detection by PCR-RFLP and direct sequencing. (A) Electrophoretic patterns following Hin6 I digestion. PCR products were 173 bp. Only amplicons with the wild-type sequences were digested, yielding two bands of 143 bp and 30 bp. The digestion products were separated by 2% agarose gel electrophoresis. M, DNA marker with 50-bp ladder. Lanes 1, 2, and 4–6, normal individuals. Lane 3, heterozygous individual for the variant. (B) Chromatograms obtained by sequencing. Left, wild-type; Right, heterozygote.

Seventeen of the 34 probands (50%) carried the same mutation, c.565C>T (p.Arg189Trp), in exon 7. The recurrent mutation was then evaluated in an independent case-control study by PCR/RFLP (Figure 3). The genotyping data from the RFLP assays were completely consistent with those obtained by sequencing. The demographic data and acquired thrombotic risk factors of all subjects are presented in Table 2. The heterozygous c.565C>T mutation was identified in 59 (5.88%) venous thrombosis patients and 9 (0.87%) controls. The homozygous genotype was absent in all subjects. As illustrated in Table 3, the observed OR of this mutation was 6.91 (95%CI = 3.42–13.98; *P* = 4.67×10^−10^). Multivariate logistical regression analysis revealed that the association between the variant and VT was still significant (OR = 7.34; 95%CI = 3.61–14.94; *P* = 3.88×10^−8^) after adjustment for age, gender, smoking status, alcohol abuse, malignant tumor, type 2 diabetes, sedentariness/immobilization, and pregnancy/puerperium in the dominant model. Meanwhile, this mutation was also detected and confirmed in 4 of the 492 (0.81%) healthy participants from Shanghai, indicating that this mutation is not restricted to the central Chinese population and is likely to be prevalent in the general population.

**Table 2:** Data from participants enrolled in the secondary case-control study.

Variable	Cases		Controls		*P*
	No.	%	No.	%	
Age (years)	51.45±14.36		50.26±14.52		0.063
Sex					0.970
*Male*	532	53.04%	546	52.96%	
*Female*	471	46.96%	485	47.04%	
Smoking					0.009
*Yes*	227	22.6%	185	17.9%	
*No*	776	77.4%	846	82.1%	
Alcohol drinker					0.717
*Yes*	117	11.7%	115	11.2%	
*No*	886	88.3%	916	88.8%	
Malignant tumor					1.207×10^−13^
*Yes*	65	6.5%	5	0.5%	
*No*	938	93.5%	1026	99.5%	
T2DM					0.037
*Yes*	42	4.2%	26	2.5%	
*No*	961	95.8%	1005	97.5%	
Sedentariness					0.031
*Yes*	23	2.3%	11	1.1**%**	
*No*	980	97.7%	1020	98.9**%**	
Pregnancy/puerperium					
*Yes*	26	2.6%	14	1.4%	0.045
*No*	977	97.4%	1017	98.6%	

Years of age were expressed as mean±standard deviation. Age of cases: age at the first incident. Age of controls: age at enrollment. Age was further divided into three levels: under 40 years, 40 to 60 years, and over 60 years. Sedentariness/immobilization reflects the 4 weeks prior to the venous thrombosis incident. A Chi-squared test was used to compare the differences between groups according to gender, smoking status, alcohol abuse, malignant tumor, type 2 diabetes, sedentariness/immobilization, and pregnancy/puerperium. A Student's *t* test was used to compare the mean ages of the cases and controls. VT = venous thrombosis. T2DM = type 2 diabetes mellitus.

**Table 3:** Association between the *PROC* c.565C>T and venous thrombosis in the Chinese population.

*PROC* c.565C>T	Cases		Controls		Without adjustment			After adjustment*		
	No.	%	No.	%	OR	95%CI	*P*	OR	95%CI	*P*
C/C	944	94.12%	1022	99.13%	1	-	-	1	-	-
C/T	59	5.88%	9	0.87%	7.10	3.50–14.39	3.31×10^−10^	7.34	3.61–14.94	3.88×10^−8^
MAF	-	2.94%	-	0.44%	6.91	3.42–13.98	4.67×10^−10^	-	-	-

Test for H-W equilibrium in controls: *P* = 0.89. CI = confidence interval. MAF = minor allele frequency.

* Data were analyzed by logistic regression adjusted for age, gender, smoking status, alcohol abuse, malignant tumor, type 2 diabetes, sedentariness/immobilization, and pregnancy/puerperium.

### Thrombotic risk in first-degree relatives with the c.565C>T mutation

As shown in Table 4, the incidence of thrombosis was greater in first-degree relatives with the c.565C>T mutation (7.9 per 1000 person-years) compared with those without this mutation (0.8 per 1000 person-years), with a relative risk of 8.8 (95%CI = 1.1–71.6) for thrombosis.

**Table 4:** Family analysis of thrombotic risk in c.565C>T carriers and non-carriers.

	Carriers	Non-carriers
Number	20	29
Male/Female	12/8	16/13
Age at first thrombosis, median (range)	51.5 (49–61)	51
Years of follow up	890	1264
Number of VT events	7	1
Events per 1000 person-years (95%CI)	7.9 (7.6–8.1)	0.8
Relative risk for thrombosis (95% CI)*	8.8 (1.1–71.6)	1

* Data were analyzed using a Cox regression model adjusted for age and gender.

## Discussion

Protein C deficiency is known to be associated with an increased risk of VT and its genetic background has been analyzed in several populations [29]–[31]. In the present study, we systematically investigated the genetic characteristics of PC deficiency in the Chinese population.

A total of 18 different mutations, including one recurrent mutation, were identified in 34 probands. Six of the detected variants (c.400+5G>A, p.Phe181Val, p.Arg189Trp, p.Arg211Gln, p.Arg220Trp, and p.Ser312Leu) have been reported previously as disease-causing mutations [29], [32]–[34].

Eight novel coding sequence variants contributed to seven amino acid exchanges. The variant p.Lys70Glu is located in the Gla domain, which is involved in the interaction between PC and anionic phospholipid surfaces [35]. This alteration creates a new Glu residue and changes the charge nature of this site, which might reduce the efficiency of the interaction with phospholipid membranes. The p.Cys106Gly and p.Cys175Tyr variants eliminate Cys residues that are important for disulfide bond formation in the EGF-1 and EGF-2 domains (Figure 2A–2B), respectively. These mutations might result in the production of an aberrant multimer or high molecular weight complex [36]. Variants in the enzymatic serine protease domain (p.Ser223Arg, p.Asp297His, p.Glu327Val, and p.Val420Leu) are located adjacent to either the serine protease active site triad or the substrate binding region (Figure 2C–2H). These substitutions may impair the structural integrity and stability of the heavy chain and attenuate the catalytic activity of the protein [37].

The three novel null mutations (c.237+5G>A, c.349_352del, c.719dupG) are evidently detrimental because they either impair mRNA splicing by disrupting the consensus sequences of the exon-intron boundary or produce a premature stop codon. Abnormal translation products may not be stable *in vivo* due to nonsense-mediated mRNA decay [38], [39]. The c.*73C>T mutation is located in the untranslated region of the last exon. The 3′-untranslated region is essential for the posttranscriptional regulation of mRNA expression. Mutations in this region almost always reduce the stability and half-life of transcripts [40], [41].

The recurrent c.565C>T variant (p.Arg189Trp, R147W, or rs146922325:C>T) was identified in 17 probands, accounting for half of the cases of PC deficiency. The retrospective family study showed that first-degree relatives bearing this variant had an 8.8-fold increased risk of venous thrombosis. The statistical power of this analysis is approximately 0.989, with a type I error probability of 0.01. However, the confidence intervals was wide due to the small number of family members enrolled and the low incidence of thrombosis in the non-carrier group. A larger prospective study will be required to confirm our observations.

**Figure 4:**
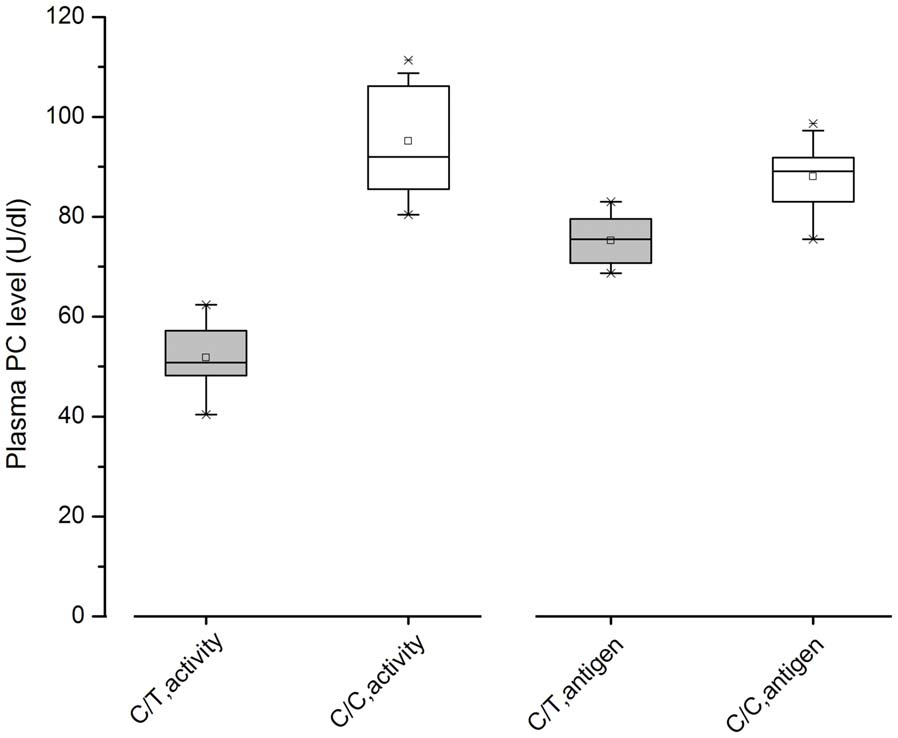
Plasma protein C level of the *PROC* c.565C>T heterozygous subjects and normal individuals. The mean, median, interquartile range, and range of protein C levels (activity and antigen) are shown. Gray box, C/T genotype (n = 17). White box, C/C wild-type (n = 20).

The R147W substitution is adjacent to the EGF-2 domain at the C-terminal of the light chain and may impair the interaction of PC with other molecular such as thrombin-thrombomodulin complex, substrate, or phospholipid [42]. Further functional studies are required to define the deleterious effect of this mutation on the activation of PC, the inactivation of FVa by APC, and the cytoprotective role of APC. This missense mutation was first described in a family with PC deficiency as well as an asymptomatic individual in 1995 [34], [43]. Consistent with previous studies, our analysis showed that the heterozygous state is associated with decreased functional activity (40.4–62.3 U/dl) and a relatively normal antigen level (68.7–83.0 U/dl, Figure 4), indicating type II PC deficiency. This variant is believed to be a rare mutation and the corresponding minor allele frequency in dbSNP is 0.000. Because its prevalence, a case-control study was further conducted. This variant was also present in 9 out of the 1031 control subjects (0.87%) and was significantly associated with VT risk. The statistical power of our analysis is approximately 0.999, with a type I error probability of 0.01. In combination with the genotyping data from the 492 DNA samples from Shanghai, the c.565C>T variant was present in approximately 0.85% (95%CI = 0.38%–1.31%) of the general population. The carrier rate of this variant allele is relatively lower than that of factor V Leiden (5%–10%) and prothrombin G20210A (2%–4%), two polymorphisms that confer moderate predisposition to VT in Whites [44]. However, it is more common than the antithrombin Cambridge II variant (approximately 0.2%) [45] and other rare but severe anticoagulant protein (AT, PC, and PS) mutations. One previous study [46] showed that the estimated prevalence of protein C deficiency in the general Chinese population is approximately 0.29%. In their study, only subjects with PC activity below the 1^st^ percentile were selected for DNA mutation analysis and considered to have “PC deficiency”. Subjects with a PC level between the 1^st^ and 2.5^th^ percentiles might also have a genetic defect. Therefore, these genetic variants might have been missed. The *PROC* c.565C>T was one such of the missed mutations. When this moderate mutation is taken into account, however, it is clear that PC deficiency has been underestimated and may be more frequent in the Chinese population than in Japanese or European populations [47], [48].

In conclusion, we investigated the genetic background of PC deficiency in the Chinese population for the first time and identified one recurrent variant as well as 17 other mutations. The NM_000312.3:c.565C>T mutation is not only the most frequent variant for PC deficiency but also a significant risk factor for venous thrombosis in Chinese individuals. Although this variant was identified about 16 years ago as a rare mutation in Western populations, its high prevalence and potential clinical significance in the Chinese population was not appreciated until now. The prevalence of and the thrombotic risk associated with this mutation in other populations (especially other Asian populations) should be further evaluated. This genetic detection may be included in the routine thrombophilia screening, and carriers of this variant may benefit from early diagnosis and better prevention [49].
